# Chemotherapy-Induced Constipation and Diarrhea: Pathophysiology, Current and Emerging Treatments

**DOI:** 10.3389/fphar.2016.00414

**Published:** 2016-11-03

**Authors:** Rachel M. McQuade, Vanesa Stojanovska, Raquel Abalo, Joel C. Bornstein, Kulmira Nurgali

**Affiliations:** ^1^Centre for Chronic Disease, College of Health and Biomedicine, Victoria University, MelbourneVIC, Australia; ^2^Área de Farmacología y Nutrición, Universidad Rey Juan CarlosMadrid, Spain; ^3^Grupo de Excelencia Investigadora URJC, Banco de Santander Grupo Multidisciplinar de Investigación y Tratamiento del Dolor, Universidad Rey Juan CarlosMadrid, Spain; ^4^Unidad Asociada al Instituto de Química Médica del Consejo Superior de Investigaciones CientíficasMadrid, Spain; ^5^Unidad Asociada al Instituto de Investigación en Ciencias de la Alimentación del Consejo Superior de Investigaciones CientíficasMadrid, Spain; ^6^Department of Physiology, University of Melbourne, MelbourneVIC, Australia

**Keywords:** chemotherapy, chemotherapy-induced constipation, chemotherapy-induced diarrhea, pathophy-siology, treatments

## Abstract

Gastrointestinal (GI) side-effects of chemotherapy are a debilitating and often overlooked clinical hurdle in cancer management. Chemotherapy-induced constipation (CIC) and Diarrhea (CID) present a constant challenge in the efficient and tolerable treatment of cancer and are amongst the primary contributors to dose reductions, delays and cessation of treatment. Although prevalence of CIC is hard to estimate, it is believed to affect approximately 16% of cancer patients, whilst incidence of CID has been estimated to be as high as 80%. Despite this, the underlying mechanisms of both CID and CIC remain unclear, but are believed to result from a combination of intersecting mechanisms including inflammation, secretory dysfunctions, GI dysmotility and alterations in GI innervation. Current treatments for CIC and CID aim to reduce the severity of symptoms rather than combating the pathophysiological mechanisms of dysfunction, and often result in worsening of already chronic GI symptoms or trigger the onset of a plethora of other side-effects including respiratory depression, uneven heartbeat, seizures, and neurotoxicity. Emerging treatments including those targeting the enteric nervous system present promising avenues to alleviate CID and CIC. Identification of potential targets for novel therapies to alleviate chemotherapy-induced toxicity is essential to improve clinical outcomes and quality of life amongst cancer sufferers.

## Introduction

Cancer is a leading cause of death worldwide (Jemal et al., 2011; Torre et al., 2015) with approximately 14.1 million new cancer cases and 8.2 million cancer deaths in 2012 alone (Ferlay et al., 2015). Although advances in modern medicine have improved scanning and cancer detection techniques, the burden for global health of cancer is expected to intensify in decades to come particularly in low and middle income families and economically developed countries (Jemal et al., 2010a,b). Population aging and growth coupled with the adoption of high risk lifestyle choices such as smoking, physical inactivity, and westernization of diets have been identified as underlying factors contributing to the increasing incidence of cancer worldwide (Jemal et al., 2011). It is now anticipated that by 2025 more than 20 million people will be affected by cancer (Ferlay et al., 2015).

Most cancer patients receive curative or palliative chemotherapeutic intervention throughout the course of treatment (Louvet et al., 2002; Benson et al., 2004b; Kaufmann et al., 2006; Wagner et al., 2006; Goffin et al., 2010; Okines et al., 2010). Although chemotherapy has greatly improved overall survival in many types of cancer, cytotoxic side-effects are a significant hurdle greatly impeding the clinical application of otherwise beneficial therapies (Xue et al., 2011; Iwamoto, 2013). GI side-effects such as nausea, vomiting, ulceration, bloating, constipation and, in particular, diarrhea are major obstacles causing delays, adjustments, and discontinuation of treatment whilst greatly impacting quality of life in many cancer patients (Benson et al., 2004a; Stringer et al., 2007, 2009d; Denlinger and Barsevick, 2009; Peterson et al., 2011). Although specific chemotherapeutic agents have been correlated with heightened incidence of GI side-effects (**Table 1**), incidences as high as 40% in patients receiving standard dose chemotherapy and 100% in patients receiving high dose chemotherapy have been reported (McQuade et al., 2014). Furthermore, the incidence of chronic post-treatment constipation and diarrhea amongst cancer survivors has been estimated to be as high as 49% with episodes persisting up to 10 years after the cessation of treatment (Schneider et al., 2007; Denlinger and Barsevick, 2009; Kim et al., 2012). The underlying mechanisms of CIC and diarrhea (CID) remain unclear. Although mucositis presenting as inflammation and ulceration of the intestinal epithelium is a significant contributing factor, the pathophysiology of CID and CIC is likely to be complex, involving several overlapping inflammatory, secretory and neural mechanisms.

**Table 1:** Gastrointestinal side-effects of chemotherapy.

Mechanisms	Chemotherapeutic agents	Cancer type	GI side-effects
Alkylating Agents	Cisplatin	Lung, Breast, Stomach, Colorectal, Liver	Nausea, Vomiting, **Diarrhea, Constipation** (Ilson et al., 1999; Ardizzoni et al., 2007)
	Cyclophosphamide	Breast	Nausea, Vomiting, Abdominal Pain, **Diarrhea** (Fraiser et al., 1991; Boussios et al., 2012)
	Oxaliplatin	Colorectal, Breast, Stomach	Nausea, Vomiting, **Diarrhea, Constipation** (Extra et al., 1990; Kim et al., 2003)
Antimetabolites	5-Fluorouracil	Breast, Colorectal, Stomach, Liver	Nausea, Vomiting, Abdominal Pain, **Diarrhea** (Douillard et al., 2010; Boussios et al., 2012)
	Capecitabine	Colorectal, Breast, Stomach	Nausea, Vomiting, **Diarrhea** (Walko and Lindley, 2005; Stathopoulos et al., 2007; Boussios et al., 2012)
	Gemcitabine	Lung, Breast	Nausea, Vomiting, Abdominal Pain, **Constipation, Diarrhea** (Wolff et al., 2001; Mutch et al., 2007; Boussios et al., 2012)
	Methotrexate	Breast	Nausea, Vomiting, Abdominal Pain, **Diarrhea** (Boussios et al., 2012)
Anthracycline	Doxorubicin	Breast, Lung, Liver	Nausea, Vomiting, Abdominal pain, GI Ulceration, **Diarrhea** (Boussios et al., 2012; Tacar et al., 2013)
Immunomodulating agent	Thalidomide	Myeloma, Kidney	Nausea, Vomiting, **Diarrhea, Constipation** (Smith et al., 2008)
Mitotic inhibitors	Cabazitaxel	Prostate	Nausea, Vomiting, Abdominal pain, **Diarrhea** (Nightingale and Ryu, 2012; Dieras et al., 2013)
	Docetaxel	Prostate, Breast, Lung, Stomach	Nausea, Vomiting, **Diarrhea** (Boussios et al., 2012)
	Paclitaxel	Lung, Stomach, Prostate, Breast	Nausea, Vomiting, **Diarrhea** (Boussios et al., 2012)
	Vincristine	Breast, Lung	**Constipation**, Abdominal Pain (Holland et al., 1973)
Topoisomerase inhibitor	Irinotecan	Colorectal, Breast, Stomach, Lung	Nausea, Vomiting, Acute and Delayed **Diarrhea** (Hecht, 1998)

## Chemotherapy-Induced Diarrhea

Diarrhea is a frequently under-recognized clinical issue that significantly affects morbidity and mortality of cancer patients worldwide (Maroun et al., 2007). Prevalence and severity of CID vary greatly depending on chemotherapeutic regime administration and dosage. A direct correlation between cumulative dose and severity of CID has been recognized, with high dose regimens associated with heightened incidence of CID (Verstappen et al., 2003). Certain regimens, especially those containing 5-fluorouracil and irinotecan are associated with rates of CID of up to 80% (Benson et al., 2004a; Richardson and Dobish, 2007) with one third of patients experiencing severe (grade 3 or 4) diarrhea (**Table 2**) (Maroun et al., 2007).

**Table 2:** Common toxicity criteria for diarrhea and constipation grading (adapted from the National Cancer Institute).

Toxicity	Grade 1	Grade 2	Grade 3	Grade 4	Grade 5
Diarrhea	Increase of <4 stools per day over baseline.	Increase of 4–6 stools per day over baseline.	Increase of >7 stools per day over baseline. Incontinence. Hospitalization.	Life threatening consequences. Urgent intervention indicated.	Death
Constipation	Occasional or intermittent symptoms; occasional use of stool softeners, laxatives, dietary modification, or enema.	Persistent symptoms with regular use of laxatives or enemas indicated.	Symptoms interfering with activities of daily living; obstipation with manual evacuation indicated	Life-threatening consequences (e.g., obstruction, toxic megacolon).	Death

Chemotherapy-induced diarrhea severely interferes with anti-cancer treatment, resulting in treatment alterations in approximately 60% of patients, dose reductions in 22% of patients, dose delays in 28% of patients and complete termination of treatment in 15% of patients (Arbuckle et al., 2000; Dranitsaris et al., 2005). Moreover, CID has been reported to last as long as 10 years post-treatment (Denlinger and Barsevick, 2009). Persistent and severe chemotherapy-associated Diarrhea is correlated with significant malnutrition and dehydration resulting in concomitant weight loss (cachexia), fatigue, renal failure, hemorrhoids, and perianal skin breakdown (Mitchell, 2006; Shafi and Bresalier, 2010). CID related dehydration is linked to early death rates in roughly 5% of patients undergoing anti-cancer treatment (Rothenberg et al., 2001). Further to this, chemotherapeutic administration may also prompt severe intestinal inflammation, bowel wall thickening and ulceration (Kuebler et al., 2007) contributing to clinical disruptions with potentially life-threatening ramifications (Rothenberg et al., 2001; Benson et al., 2004a; Stein et al., 2010).

For over 30% of CID sufferers it interferes with their daily activities (Stein et al., 2010), with detrimental effects on the mental and social health of cancer survivors. Persistent and uncontrollable CID has been linked to anxiety, depression, social isolation, and low self-esteem (Viele, 2003), emphasizing the importance of both elucidating the underlying mechanisms of CID and improving treatment efficacy (Carelle et al., 2002).

### Pathophysiology of Chemotherapy-Induced Diarrhea

Although several chemotherapy regimens have been associated with Diarrhea to varying degrees (**Table 1**), most basic research into the mechanisms underlying CID has focused on irinotecan and its active metabolite SN38 (Gibson and Keefe, 2006). As diarrhea is a well-recognized side-effect of irinotecan treatment, the histological changes that occur throughout the GI tract in response to irinotecan administration have been examined in several animal studies (Araki et al., 1993; Ikuno et al., 1995; Takasuna et al., 1996; Gibson et al., 2003). Pronounced crypt ablation, villus blunting and epithelial atrophy in the small and large intestines have been reported (Logan et al., 2008), resulting in mucosal damage and degeneration being a major theme throughout the literature surrounding CID. Although patients do not routinely have imaging or endoscopy to diagnose the chemotherapy-induced mucosal inflammation (Touchefeu et al., 2014), CID is still largely believed to be a form, or by-product, of GI mucositis. Mucositis is defined as mucosal injury presenting as inflammation and ulceration, resulting in alterations of intestinal microflora and GI secretion (Stringer, 2009; Stringer et al., 2009a,b). The basic pathophysiology of mucositis can be broken into 5 sequential phases: (i) initiation; (ii) up-regulation; (iii) signaling and amplification; (iv) ulceration and inflammation; and (v) healing (Sonis et al., 2004; Lee et al., 2014).

Initiation of mucositis is believed to result from direct or indirect effects of cytotoxic chemotherapeutics on the rapidly dividing epithelial cells in GI tract, triggering apoptosis. This leads to reductions in crypt length and villus area, coupled with activation of nuclear factor-kappa B (NFκB) and subsequent up-regulation of pro-inflammatory cytokines including interleukin 1 (Lawrence, 2009), which contribute to ulceration and inflammation in the mucosal epithelium (Gibson et al., 2003; Stringer et al., 2007, 2008, 2009a,d; Logan et al., 2008). Intestinal microbiota is known to play an integral role in intestinal homeostasis and are now believed to play a key role in the development of mucositis (van Vliet et al., 2010; Touchefeu et al., 2014). Recent studies have revealed that chemotherapeutic administration has effects on intestinal microbial composition (Stringer et al., 2009a,b), and fecal microbiota (Touchefeu et al., 2014).

Much of the research investigating the effects of chemotherapeutic administration on microbiota has focused primarily on topoisomerase I inhibitor, irinotecan, due to the involvement of microbiota in its metabolism (Stringer, 2013). Upon metabolism in the liver, irinotecan is converted to its active metabolite SN-38 by enzyme carboxylesterase, before being deactivated through glucuronidation by uridine diphosphate glucuronosyltransferase 1A1 (UGT1A1) to form SN38 glucuronide (SN38-G). However, SN38G may be reactivated to SN38 in the presence of enzyme β-glucuronidase, which may be produced by the intestinal microbiome. Several studies have shown a shift in commensal bacteria, in particular *Bifidobacterium* spp. toward *Salmonella* spp. and *Escherichia coli* following irinotecan administration (Stringer et al., 2009b). Of the β-glucuronidase-producing bacteria, *Bacteroides* spp. has been shown to decrease following irinotecan treatment, concurrently *Staphylococcus* spp*., Clostridium* spp. and *E. coli* have been found to be increased, whilst presence of beneficial bacteria, *Lactobacillus* spp. and *Bifidobacterium* spp. was decreased following irinotecan treatment (Stringer et al., 2007). When given in combination with antimetabolite 5-fluorouracil, both *Clostridium cluster XI* and *Enterobacteriaceae* presence was found to be increased, whilst treatment with 5-fluorouracil alone has also been found to increase the presence of *Clostridium* spp. and *Staphylococcus* spp. at 24 h post-treatment (Stringer et al., 2009c).

These changes in microbiota are believed to play an important role not only in maintaining intestinal homeostasis and integrity but in the modulation of inflammatory responses through interaction with Toll-like receptors and the nucleotide oligomerization domain receptors that activate NFκB (van Vliet et al., 2010). In the healing phase, proliferation and differentiation of the GI epithelium return approximately 2 weeks post-chemotherapy (Sonis et al., 2004; Lee et al., 2014), but functional changes persist after recovery of morphological changes (Keefe et al., 2000; Rubenstein et al., 2004). The pathophysiology underlying these persistent changes in GI functions includes several overlapping secretory, osmotic, inflammatory, and neurogenic mechanisms (McQuade et al., 2014).

Disruption to water and electrolyte balance within the GI tract is a key component in the pathophysiology of all types of diarrhea. Direct mucosal damage has been suggested as a major contributor to malabsorption and hypersecretion associated with CID (Richardson and Dobish, 2007; Stringer et al., 2007, 2009b; Stein et al., 2010). Studies using animal models of CID have demonstrated increased apoptosis in the crypts of both the jejunum and colon, resulting in metaplasia of goblet cells and excessive mucous secretion (Ikuno et al., 1995; Gibson et al., 2003). Hyperplasia of the rapidly dividing crypt cells in the epithelium of the gut probably results in heightened proportions of immature secretory cells, leading to increased secretion and decreased absorptive capacity of the villi, thereby contributing to the onset of diarrhea (Castro-Rodríguez et al., 1997). Retention of non-absorbable compounds within the lumen triggers an osmotic shift of water into the lumen (Castro-Rodríguez et al., 1997; Richardson and Dobish, 2007; Stringer et al., 2007). This reduced absorptive capacity and increased secretion in the small intestines results in increased fluid and solutes in the intestinal lumen and overwhelms the absorptive capacity of the colon resulting in diarrhea (Gibson and Keefe, 2006).

Secondary to mucosal damage, CID has been associated with mucosal inflammation throughout the GI tract (Logan et al., 2008). Increased expression of cyclooxygenase (COX)-2, associated with increased release of prostaglandin E2 (PGE2), is seen in rat colon following irinotecan administration (Yang et al., 2005). PGE2 stimulates colonic secretion and hyperperistalsis of the gut, whilst inhibiting sodium, potassium and adenosine triphosphatase, and triggering excessive chloride secretion, all of which further contribute to the onset of diarrhea (Kase et al., 1997a,b; Leahy et al., 2002; Yang et al., 2005). Further, irinotecan stimulates the production of thromboxane A2, a potent physiological stimulant of chloride and water secretion in the colon (Sakai et al., 1997; Suzuki et al., 2000) as well as tumor necrosis factor- α (TNF-α) a pro-inflammatory cytokine and a primary mediator of immune regulation associated with CID (Yang et al., 2005).

Chemotherapy can induce damage to the ENS (Vera et al., 2011; Wafai et al., 2013) which may also underlie GI secretory disturbances involved in pathophysiology of CID. Innervation of the GI tract is primarily from the ENS, sometimes referred to as “the second brain” due to its ability to function autonomously of the central nervous system (Phillips and Powley, 2007). The ENS is comprised of ganglia, primary interganglionic fiber tracts as well as secondary and tertiary fibers which project to many of the effector systems of the gut including muscle cells, glands, and blood vessels (Hansen, 2003). The ENS is divided into two major ganglionated plexi, the myenteric (Auerbach’s), and submucosal (Meissner’s), which are responsible for controlling gut functions including motility, secretion, absorption and vascular tone. Enteric neuropathy has been linked to a variety of GI pathologies, in part due to its regulation of intestinal epithelial function and colonic motility (De Giorgio et al., 2000, 2004; De Giorgio and Camilleri, 2004; Chandrasekharan et al., 2011; Furness, 2012). However, effects of chemotherapeutics on enteric neurons and GI dysfunction have been largely overlooked until recently. It has been shown that chronic treatment with cisplatin results in myenteric neuronal loss, increase in amplitude of the neurally induced contractions of the gastric fundus strips in mice and occasional diarrhea (Pini et al., 2016). Thus enteric neuropathy may be an underlying cause of chemotherapy-induced GI dysmotility.

Movement of fluid between the lumen of the intestine and the body fluid compartments is a complex and tightly regulated process involving neural, endocrine, paracrine, and autocrine systems that act via the enteric neurons within the submucosal plexus (Lundgren et al., 2000; Johnson et al., 2012). Situated superficially to the mucosa, the submucosal plexus lies between the circular muscle and muscularis mucosa layer of the mucosa and derives innervation from neurons in the myenteric plexus as well as direct innervation from branches of the sympathetic and parasympathetic nervous systems. The submucosal plexus innervates the mucosal epithelium and submucosal arterioles to control and maintain water and electrolyte balance, secretion and vascular tone (Furness, 2012). Fluid is absorbed from the lumen containing nutrients via ion-coupled transporters and returned through secretomotor reflexes. Through activation of secretomotor neurons, water and electrolytes are moved from the interstitium of the lamina propria to the lumen, drawn from both the circulation and the absorbed fluids. Neural control of secretion and absorption of water and electrolytes occurs on multiple interacting levels. While there are secretomotor circuits confined to the submucosal plexus, they can be directly controlled by circuitry within the myenteric plexus. Despite the important role of the ENS in controlling secretory function, very little research has been undertaken to elucidate the relationship between the ENS and CID. Enteric neuropathy and/or neuronal dysfunction may be a contributing factor in chemotherapy-induced secretory dysfunction.

### Current Treatments for Chemotherapy-Induced Diarrhea

Chemotherapy-induced diarrhea may be classified as uncomplicated (grade 1–2 with no complications) or complicated (grade 3–4 with one or more complicating signs or symptoms), early onset (<24 h after administration) or late onset (>24 h after administration) and may be categorized as persistent (present for >4 weeks) or non-persistent (present for <4 weeks) according to the National Cancer Institute’s Common Terminology Criteria for Adverse Effects grading system (Stein et al., 2010). Although uncomplicated CID may be managed by modification of the diet and administration of standard anti-diarrheal drugs such as loperamide, octreotide and tincture of opium, complicated diarrhea requires aggressive high dose anti-diarrheal administration and hospitalization (McQuade et al., 2014). The recommendations on the management of CID were published in 1998 and updated in 2004 (Wadler et al., 1998; Benson et al., 2004a), providing guidelines for evaluation and management of CID. These guidelines have not been updated since 2004. Currently the only drugs recommended in the updated treatment guidelines are opioid derivatives such as loperamide and deodorized tincture of opium (DTO), and octreotide.

#### Loperamide

Loperamide is a non-analgesic agonist that acts at μ-opioid receptors at the level of the myenteric plexus to decrease intestinal motility (Regnard et al., 2011). High dose loperamide alleviates diarrhea associated with chemotherapeutic administration (Stein et al., 2010). However, its use leads to a range of side-effects including severe constipation, abdominal pain, dizziness, rashes as well as worsening of already present bloating, nausea and vomiting (Lenfers et al., 1999; Stein et al., 2010). High dose loperamide is reported to increase incidents of paralytic ileus, in association with abdominal distension (Sharma et al., 2005; Richardson and Dobish, 2007). Despite these severe side-effects, loperamide remains the standard first line therapy for CID.

#### Octreotide

Octreotide is a synthetic somatostatin analog that promotes absorption by inhibiting specific gut hormones to increase intestinal transit time (Högenauer et al., 2002; Mitchell, 2006) as well as hyperpolarizing enteric secretomotor neurons (Högenauer et al., 2002). Octreotide is administered to treat both complicated diarrhea and loperamide-refractory diarrhea and is generally reserved as a second line treatment for patients who are unresponsive to loperamide after 48 h, despite loperamide dose escalation (Regnard et al., 2011). Although octreotide decreases CID effectively, severe side-effects including slow and/or uneven heartbeat, severe constipation, stomach pain, enlarged thyroid, vomiting, nausea, headache and dizziness occur in over 10% of patients (Bhattacharya et al., 2008).

#### Deodorised Tincture of Opium

Deodorized tincture of opium (DTO) is another widely used antidiarrheal agent, despite the absence of literature to support its use in CID treatment (Stein et al., 2010). Similar to loperamide, DTO activates μ-opioid receptors within the GI tract inhibiting intestinal peristalsis, increasing intestinal transit time and promoting fluid reabsorption (Richardson and Dobish, 2007). The efficacy of DTO in treatment of CID has not been reported, however, it is a commonly used anti-diarrheal drug and may be considered as a second-line therapy for persistent and uncomplicated diarrhea (Richardson and Dobish, 2007). DTO contains 10 mg/ml of morphine and is one of the most potent forms of orally administered morphine available by prescription. DTO induces many side-effects including euphoria, nausea, vomiting, painful/difficult urination, stomach and abdominal pain, seizures and allergic reactions. Further, DTO administration associates with psychological and physical dependence, miosis, respiratory depression (Benson et al., 2004a; Richardson and Dobish, 2007) and constipation, with continued/prolonged opioid use linked to severe constipation (Benyamin et al., 2008).

## Chemotherapy-Induced Constipation

Constipation is a frequent, and underestimated, complication in patients with advanced cancer (Mancini and Bruera, 1998). As constipation is a subjective sensation, there is difficulty surrounding acceptance of a universal definition, although it is broadly recognized clinically as a mixture of reduced frequency of bowel action and increased stool consistency (Connolly and Larkin, 2012). Constipation occurs in 50–87% of advanced cancer patients (Abernethy et al., 2009). Constipation is the third most common symptom in patients receiving cytotoxic chemotherapy with an overall prevalence of 16%, with 5% classified as severe and 11% classified as moderate (Yamagishi et al., 2009; Anthony, 2010).

The mechanisms underlying CIC are poorly defined with minimal clinical studies existing. Distinguishing true CIC from secondary constipation from drugs given to control other chemotherapy or cancer-induced symptoms (such as anti-emetics for nausea and vomiting and opioids for pain) is a major issue hindering investigation (Gibson and Keefe, 2006). Given the scarcity of literature concerning CIC it is hard to estimate accurate incidence and severity among all chemotherapy-treated cancer sufferers, but specific chemotherapeutic agents such as thalidomide, cisplatin and vinca alkaloids such as vincristine, vinblastine, and vinorelbine induce true CIC in up to 80–90% of patients (Ghobrial and Rajkumar, 2003; Pujol et al., 2006; Stojanovska et al., 2015).

Constipation is not deemed to be of clinical importance until it causes physical risks or impairs quality of life. Constipation can cause a number of significant symptoms. Severely constipated patients experience abdominal distension usually accompanied by severe abrupt episodes of abdominal pain (Falcón et al., 2016). Furthermore, rectal tearing, hemorrhoids and rectal fissures caused by passing hard, dry stool are frequent complications of constipation (Leung et al., 2011). Untreated constipation may progress to obstipation, severe persistent constipation, which can have life threatening complications associated with fecal impaction and bowel obstruction (Leung et al., 2011). Fecal impaction, the presence of unpassable masses of stool, and increases intraluminal pressure within the bowel can lead to ischaemic necrosis of the mucosa, pain, bleeding, and perforation. Fecal impaction is also well recognized as a factor in urinary incontinence in the elderly (MacDonald et al., 1991). Constipation can also cause confusion, increase retroperitoneal or liver pain, trigger rapid onset nausea with or without vomiting in the presence of intestinal blockage and lead to inadequate absorption of oral drugs (Mancini and Bruera, 1998), greatly affecting the tolerability and efficacy of chemotherapeutic administration. There is accumulating evidence that self-reported constipation and functional constipation lead to significant impairment of quality of life, with the implication that this is a serious condition in the majority of people aﬄicted (Talley, 2003; Dennison et al., 2005), however, little work has been undertaken to elucidate prevalence and mechanisms.

### Pathophysiology of Chemotherapy-Induced Constipation

Normal bowel function requires the coordination of motility, mucosal transport, and defecation reflexes (Mancini and Bruera, 1998). Broadly constipation can be classified into three categories: normal-transit constipation, defecatory disorders and slow-transit constipation (Lembo and Camilleri, 2003). Normal-transit constipation is the most common form of constipation, where frequency of colonic evacuation is normal, yet patients believe they are constipated due to a perceived difficulty with evacuation or the presence of hard stools. Symptoms of normal-transit constipation include bloating and abdominal pain or discomfort, as well as increased psychosocial distress (Ashraf et al., 1996). Constipation resulting from defecatory disorders is most commonly due to dysfunction of the pelvic floor or anal sphincter. Defecatory disorders may result from prolonged avoidance of the pain associated with the passage of a large, hard stool or painful, anal fissure or hemorrhoid (Loening-Baucke, 1996). Structural abnormalities, such as rectal intussusception, rectocele, obstructing sigmoidocele, and excessive perineal descent, are less common causes of defecatory disorders (Lembo and Camilleri, 2003). Slow-transit constipation is associated with infrequent urge to defecate, bloating, and abdominal pain or discomfort.

Though little clinical research has been undertaken to elucidate the underlying pathology in CIC, it has been hypothesized that CIC may result from effects of chemotherapy on nerve endings in the gut (Ghobrial and Rajkumar, 2003). The GI tract is innervated by the ENS together with fibers from extrinsic sympathetic, parasympathetic (vagus nerve) and sensory afferent neurons (Phillips and Powley, 2007). Both the extrinsic and intrinsic innervation play an important role in the motor activity of the GI tract. The internal circular smooth muscle layer and the external longitudinal smooth muscle are controlled by two main mechanisms: non-neural pacemaker cells, interstitial cells of Cajal (ICCs), which generate myogenic activity and enteric neurons which provide neurogenic supply. Neuronal terminals are closely associated with ICCs which are linked to smooth muscle cells via gap junctions. Within the ENS, three main neuronal classes of myenteric neurons govern the complex motor reflex pathways: sensory neurons, interneurons, and motor neurons. The integration of inputs from these neurons and ICCs to smooth muscle cells in the colon allows expression of various motor patterns including phasic contractile activity and tonic contractile activity which contribute to colonic motor activity and the peristaltic reflex (Gwynne et al., 2004; Dinning et al., 2009; Huizinga and Lammers, 2009; Kuizenga et al., 2015).

Subtle changes to the ENS, not evident in conventional histological examination, have been suggested as a potential underlying mechanism for abnormal colonic motor function leading to constipation (Bassotti and Villanacci, 2011). For instance, alterations in the number of myenteric neurons expressing the excitatory neurotransmitter substance P, as well as abnormalities in the inhibitory neurotransmitters, vasoactive intestinal peptide and nitric oxide, and a reduction in the number of ICCs (Cortesini et al., 1995; Tzavella et al., 1996; He et al., 2000) have been observed in patients with slow-transit constipation. However, the effects of chemotherapeutics on ENS and GI dysfunction have been largely overlooked until recently. A study investigating the effects of 5-fluorouracil-induced dysmotility in mice uncovered myenteric neuronal loss alongside delayed GI transit and inhibition of propagating colonic contractions (McQuade et al., 2016). Similar results have been demonstrated following oxaliplatin administration in mice, and cisplatin administration in rats, where enteric neuronal loss was associated with a reduction in colonic motor activity and reduced GI transit time, respectively (Vera et al., 2011; Wafai et al., 2013). Loss of enteric neurons following administration of cisplatin and oxaliplatin has been correlated with an increase in a population of the myenteric neurons expressing neuronal nitric oxide synthase (Vera et al., 2011; Wafai et al., 2013) and changes in glial cell populations (Robinson et al., 2016). These studies emphasize the importance of enteric neuronal integrity in GI function whilst suggesting neuroprotection as a potential therapeutic pathway for the treatment of chemotherapy-induced GI disorders.

### Opioid-Induced Constipation

As previously mentioned, a major limitation in the estimation and evaluation of true CIC is the onset of secondary constipation, namely opioid-induced constipation produced by opioid analgesia. Whilst opioid analgesics are the gold standard in pain relief for cancer patients, adverse effects such as opioid-induced bowel dysfunction (OIBD) and opioid-induced constipation (OIC) severely compromise their therapeutic potential (Gonzalez and Halm, 2016). Incidence of OIC ranges from 50 to 87% in terminally ill cancer patients and is positively associated with chronic opioid treatment (Abernethy et al., 2009; Abramowitz et al., 2013). Opioid receptors are located throughout the central and peripheral nervous system and are involved in pain transmission (Camilleri, 2011). In the GI tract, μ-receptors are widely distributed throughout the ileum, stomach and proximal colon where they contribute to the control of fluid and electrolyte transport as well as motility (McKay et al., 1981; Fickel et al., 1997; Garg et al., 2016). Opioid analgesics interfere with GI motility by delaying transit, stimulating non-propulsive motility and altering GI segmentation and tone through their effects on enteric neurons (De Schepper et al., 2004; Wood and Galligan, 2004). These changes coupled with activation of mucosal sensory receptors that trigger a reflex arc facilitate excessive fluid reabsorption, resulting in OIC (Panchal et al., 2007; Camilleri, 2011).

Whilst administration of laxatives remains the first-line treatment option for OIC, this intervention alone is frequently ineffective (Gatti and Sabato, 2012). Selective μ-opioid receptor antagonists are emerging as a promising first line treatment for OIC, in particular treatment with methylnaltrexone bromide (methylnaltrexone) has been found to improve GI transit in chronically ill patients and has been recommended for use in cancer patients (Gatti and Sabato, 2012). Methylnaltrexone has demonstrated efficacy in improving opioid-induced delay in the oral–caecal transit time and inducing laxation in both healthy subjects and advanced illness patients (Culpepper-Morgan et al., 1992; Thomas et al., 2005; Thomas et al., 2008). Similarly, treatment with peripheral μ-opioid receptor antagonist Alvimopan has been found to increase the frequency of spontaneous bowel movements in non-cancer patients with opioid induced bowel dysfunction (Webster et al., 2008).

### Current Treatments for Chemotherapy-Induced Constipation

The management of constipation can be divided into general interventions and therapeutic measures. The general interventions involve increasing physical exercise, fluid intake and fiber consumption, availability of comfort, privacy and convenience during defecation as well as elimination of medical factors that may be contributing to constipation (Mancini and Bruera, 1998). Therapeutic interventions for the management of constipation, including CIC involve the administration of both oral and/or rectal bulk-forming, emollient, osmotic/saline, stimulant, and lubricant laxatives (Connolly and Larkin, 2012). Laxative compounds may fall into one of several categories depending on their mechanism of action.

#### Bulk-Forming Laxatives

Bulk-forming laxatives such as methylcellulose, psyllium, and polycarbophil most closely mimic the physiologic mechanisms involved in promoting GI evacuation. Available as natural or semisynthetic hydrophilic polysaccharides, cellulose derivatives, or polyacrylic resins, bulk forming laxatives work by either dissolving or swelling in the intestines to form a viscous liquid that provides mechanical distension. This facilitates the passage of intestinal contents by stimulating peristalsis and reducing GI transit time. Although typically recommended as initial therapy for most forms of mild constipation (Kirschenbaum, 2001), bulk-forming agents can take up to 72 h to exert their effects and therefore are not ideal for the initial management of symptomatic constipation in cancer patients (Avila, 2004; Connolly and Larkin, 2012). Bulk forming laxatives require the patients to drink extra fluids as otherwise a viscous mass may form and aggravate a partial bowel obstruction. In addition, significant allergy to these substances has been reported, and their effectiveness in severe constipation is doubtful (Klaschik et al., 2003). Though they are considered safe, some patients’ experience suggests that they may worsen symptoms, causing distension, bloating, and abdominal pain (Costilla and Foxx-Orenstein, 2014).

#### Osmotic Laxatives

Osmotic laxatives such as lactulose, sorbitol, polyethylene glycol compounds, and saline laxatives (magnesium hydroxide), attract and retain fluid within GI tract (Twycross et al., 2012). Osmotic laxatives include salts of poorly absorbable cations (magnesium), anions (phosphate, sulfate) as well as molecules that are not absorbed in the small bowel but are metabolized in the colon (lactulose and sorbitol) and metabolically inert compounds such as polyethylene glycol. The presence of these molecules in the lumen results in water retention to maintain normal osmolarity of the stool (Costilla and Foxx-Orenstein, 2014). The laxative effect of these agents depends on the extent to which they remain in the lumen with the onset between 24 and 72 h (Xing and Soffer, 2001). Adverse effects such as abdominal pain, flatulence, cramping and distension can arise shortly after ingestion, although side-effects may subside after several days of treatment, higher lactulose doses can induce bloating and colic (Ford and Suares, 2011; Costilla and Foxx-Orenstein, 2014). Excessive use of osmotic laxatives may result in hypermagnesemia, hyperphosphatemia, hypercalcemia, hypernatremia, hypokalemia, and hypoalbuminemia (Xing and Soffer, 2001; Kurniawan and Simadibrata, 2011).

#### Emollient (Stool Softener) Laxatives

Emollient laxatives, also known as stool softeners, are anionic surfactants increasing efficiency of intestinal fluids and facilitating the mixing of aqueous and fatty substances within the feces; this softens the feces allowing them to move more easily through the GI tract (Avila, 2004). Stool softeners are of little value when administered unaccompanied in the treatment of long-term constipation as they do not stimulate peristalsis and evacuation, but concurrent administration with bulk-forming agents and dietary fiber provides beneficial effect reducing straining (O’Mahony et al., 2001; Avila, 2004). Increased fluid intake essential during treatment with emollient laxatives to facilitate stool softening and so are not ideal for chronic constipation in cancer patients. Docusate is the most widely used emollient laxative produced as docusate calcium, docusate sodium, and docusate potassium. The onset of action is 1–2 days after administration but might be up to 5 days. However, docusates have been found to enhance GI or hepatic uptake of other drugs, increasing the risk of hepatotoxicity (Xing and Soffer, 2001). There is also some evidence that docusates cause significant neuronal loss in the myenteric plexus (Fox et al., 1983) and cause structural changes in the gut mucosa of humans (Xing and Soffer, 2001), but the clinical significance of this remains unclear.

#### Stimulant Laxatives

Stimulant laxatives such as diphenylmethane derivatives (phenolphthalein, sodium picosulfate, anthranoids (senna and cascara), ricinoleic acid (castor oil), and surface-acting agents directly stimulate myenteric neurons to increase peristalsis resulting in reduced net absorption of water and electrolytes from the intraluminal contents (Twycross et al., 2012). Stimulant laxatives are more potent than bulk-forming and osmotic laxatives and appear to be more effective than enemas (Dosh, 2002; Scarlett, 2004). They are amongst the most commonly administered laxatives for opioid-induced constipation (Ruston et al., 2013). Although short-term use is safe, overuse can cause dehydration and long-term ingestion may result in laxative dependence. This dependence also known as ‘laxative bowel’ is thought to result from damage to the myenteric plexus and smooth muscles cells in the colon (Xing and Soffer, 2001; Kurniawan and Simadibrata, 2011).

#### Lubricant Laxatives

Lubricant laxatives emulsify themselves into the fecal mass, coating the feces and rectum for easier passage whilst retarding colonic water absorption to simultaneously soften stool (Avila, 2004). Liquid paraffin, also known as mineral oil, is the major lubricant laxative in use although seed oils from croton and arachis are also available (Xing and Soffer, 2001). These laxatives can be administered orally or rectally and are useful for patients who complain of excess straining, but long-term use is associated with malabsorption of fat soluble vitamins and minerals, as well as anal leakage (Costilla and Foxx-Orenstein, 2014). Lubricant laxatives are not routinely recommended for long-term use due to possible inflammatory conditions such as lipoid pneumonia (Schiller, 1999).

#### Rectal Laxatives

Rectal laxatives such as bisacodyl (stimulant), sodium phosphate (saline), glycerin (osmotic), and mineral oil (lubricant) (Avila, 2004) generally accepted not to be regularly used for CIC treatment (Fallon and O’Neill, 1997), but may be necessary alongside digital stimulation for treating fecal impaction or constipation associated with neurogenic bowel dysfunction. Rectal suppository of bisacodyl (stimulant) is most commonly utilized when evacuation of soft stools is needed, while glycerin suppositories are more appropriate when a hard stool needs to be softened (Fallon and O’Neill, 1997). Acute severe constipation might require an administration of rectal laxatives by enema, however, rectal suppositories or enemas cannot be used in patients with neutropenia and thrombocytopenia (O’Mahony et al., 2001).

## Emerging and Potential Treatments for CID and CIC

As current therapies for CID and CIC have limited efficacy and a plethora of adverse effects, a search for and use of novel anti-diarrheal and laxative agents is essential to improve quality of life and chemotherapeutic efficacy for cancer patients. Several emerging and already existing therapies used for treatment of other conditions such as diarrhea predominant irritable bowel syndrome (IBS-D), constipation predominant irritable bowel syndrome (IBS-C) and chronic idiopathic diarrhea and constipation could be employed for the treatment of CID and CIC.

### Chloride Channel Inhibition and Activation

Chloride is an essential ion in intestinal secretion and absorption. Secretory diarrhea, such as that experienced in irinotecan-treated patients, results from a combination of excessive secretion and reduced absorption in the intestinal lumen (Thiagarajah and Verkman, 2012). Excessive fluid secretion is driven by active chloride secretion, followed by secondary movement of water and sodium into the intestine. Although there is a lack of selective potent inhibitors of voltage gated chloride channels, inhibition of calcium-activated chloride channels throughout the intestines successfully reduced secretion of chloride into the intestinal lumen (Thiagarajah and Verkman, 2013; Thiagarajah et al., 2015). In a mouse model of rotavirus-induced severe secretory diarrhea, inhibition of calcium-activated chloride channels with a red wine extract reduced intestinal fluid secretion, diminishing the symptoms of diarrhea (Ko et al., 2014).

Conversely, chloride channel activation has been used in the management of chronic idiopathic constipation and constipation related to irritable bowel syndrome (IBS-C). Lubiprostone is a bicyclic fatty acid derived from prostaglandin E1 that specifically activates chloride channels in the intestine, whilst having no effect on smooth muscle contraction (Jun, 2013). The underlying mechanism of lubiprostone involves stimulation of electrogenic chloride secretion though activation of chloride channel type-2 (Lacy and Levy, 2007) and cystic fibrosis transmembrane conductance regulator chloride channels (Bijvelds et al., 2009) in the apical membrane of intestinal epithelial cells. Activation of these epithelial channels results in active secretion of chloride into the intestinal lumen followed by a passive secretion of electrolytes and water increasing the liquidity of the luminal contents (June, 2013). Resulting luminal distension from increased intestinal fluid content promotes GI motility and increases intestinal and colonic transit. In healthy volunteers, daily lubiprostone delays gastric emptying, increases fasting gastric volume, reduces maximum tolerated gastric volume, and accelerates small bowel and colon transit (Camilleri et al., 2006). In randomized trials involving patients with IBS-C, lubiprostone twice daily reduced abdominal pain and increased complete spontaneous bowel movement and improved stool consistency, straining, and bloating (Schey and Rao, 2011). Currently, oral lubiprostone is approved for IBS-C at 8 μg twice daily and CIC at doses of 24 μg twice daily, but approval for CIC is limited to only women who have not responded to laxatives (Davis and Gamier, 2015). At present there are no studies investigating the efficacy of lubiprostone for CID.

### Cannabinoid Receptor Inhibition and Activation

Cannabinoids mediate their effects via binding to two main G-protein coupled receptors, CB_1_ and CB_2_, widely expressed in the GI tract (Abalo et al., 2012). Although the activity of the endocannabinoid system varies between species and different regions of the GI tract within the same species, activation of CB_1_ receptors coupled to cholinergic motor neurons has been found to inhibit excitatory neuromuscular transmission in human colonic circular muscle (Hinds et al., 2006) and inhibit colonic propulsion in mice and rat (Pinto et al., 2002; Abalo et al., 2015). In recent human trials, dronabinol, a non-selective cannabinoid receptor agonist, was found to inhibit colonic motility in both healthy subjects (Esfandyari et al., 2006, 2007) and patients with IBS-related diarrhea (IBS-D) (Wong et al., 2011). Conversely, a CB_1_ receptor inverse agonist, taranabant, has been shown to improve symptoms related to slow GI motility and abdominal pain when administered *in vivo* in mice (Fichna et al., 2013). Taranabant increased the number of bowel movements after systemic and oral administration and significantly increased fecal pellet output in mice with constipation induced by ipratropium (Fichna et al., 2013). It has been demonstrated that a low dose of a non-selective cannabinoid agonist WIN55,212-2 reduced the severity of 5-fluorouracil-induced diarrhea in rats (Abalo et al., 2016).

### Guanylate Cyclase C Activation

Guanylate cyclase C is the principal receptor for heat-stable enterotoxins and plays a major role in *E. coli*-induced secretory diarrhea (Camilleri, 2010). Enterotoxins and endogenous peptides bind to guanylate cyclase C and stimulate the production of intracellular cyclic guanosine monophosphate (cGMP). Increased levels of cGMP activate the secretion of chloride ions through the cystic fibrosis transmembrane conductance regulator. Linaclotide is a minimally absorbed 14-aminoacid peptide that selectively stimulates intestinal epithelial cell guanylate cyclase C receptors, resulting in increased intracellular and extracellular cGMP leading to accelerated stool transit and laxation (Harris and Crowell, 2007). In phase II and III placebo-controlled studies in chronically constipated and IBS-C patients, linaclotide was found to accelerate colonic transit and improve abdominal pain and symptoms of constipation (Andresen et al., 2007; Johnston et al., 2009, 2010; Lembo et al., 2010). Linaclotide is particularly interesting in that it is both a laxative and analgesic, reducing visceral hypersensitivity with very few drug interactions, it is presently licensed for chronic idiopathic constipation and IBS-C in the USA (Davis and Gamier, 2015), but no trials on CIC have been reported to date.

### Probiotics, Antibiotics, and β-glucuronidase Inhibitors

With the recognition that intestinal microbiota play key roles in the pathophysiology of mucositis and development of CID/CIC, both antibiotics and probiotics have emerged as promising therapeutic options. Administration of probiotics have been shown to prevent CID in both 5-fluorouracil and irinotecan-treated animals (Bültzingslöwen et al., 2003; Bowen et al., 2007). Similarly, a combination of *Lactobacillus rhamnosus* and fiber has been found to reduce the severity of grade 3/4 5-fluorouracil/leucovorin-induced diarrhea by 15% in a randomized study of patients treated for colorectal cancer (Österlund et al., 2007). Administration of oral antibiotics, such as fluoroquinolone, has also been recommended for aggressive treatment of CID (Benson et al., 2004a; Maroun et al., 2007).

The selective inhibition of bacterial β-glucuronidase has recently been shown to alleviate drug-induced GI toxicity in mice (Wallace et al., 2015). A low-potency β-glucuronidase inhibitor showed promise in reducing the GI toxicity associated with irinotecan in rats (Fittkau et al., 2004). Similarly, oral administration of potent bacterial β-glucuronidase inhibitors has been found to reduce the severity of irinotecan-induced toxicity (Wallace et al., 2010). In clinical trials, Kampo medicine Hangeshashinto (TJ-14) which contains baicalin, a β-glucuronidase inhibitor, has been found to successfully reduce both the incidence and duration of chemotherapy-induced oral mucositis in colorectal cancer patients when compared to placebo patients (Matsuda et al., 2015). In non-small-cell lung cancer patients TJ-14 alleviated irinotecan-induced diarrhea (Mori et al., 2003). Compared with control patients, the TJ-14-treated patients showed a significant improvement in both diarrhea grade, as well as a reduced frequency of grade 3 and 4 diarrhea (Mori et al., 2003).

## Conclusion

Chemotherapy-induced diarrhea and CIC are amongst the most common chemotherapy-induced GI toxicities, heavily contributing to treatment delays, dose reductions and in some cases cessation of anti-cancer treatment, greatly effecting management and clinical outcomes. Current treatments for CID and CIC are limited and come with a profuse amount of concomitant symptoms; however, novel therapies present a promising avenue of treatment for CID and CIC. Identification of potential targets and the development of novel treatments alleviating chemotherapy-induced toxicity are essential to improve clinical outcomes and quality of life amongst cancer sufferers.

## Author Contributions

RM: conception and manuscript writing; VS, RA, JB, and KN: critical revision of the manuscript. All authors approved final version of the manuscript to be published and agreed to be accountable for all aspects of the work in ensuring that questions related to the accuracy or integrity of any part of the work are appropriately investigated and resolved.

## Conflict of Interest Statement

The authors declare that the research was conducted in the absence of any commercial or financial relationships that could be construed as a potential conflict of interest.

The reviewer HA and handling Editor declared their shared affiliation, and the handling Editor states that the process nevertheless met the standards of a fair and objective review.

## Abbreviations

CIC: chemotherapy-induced constipation
CID: chemotherapy-induced diarrhea
ENS: enteric nervous system
GI: gastrointestinal
